# Quality, Bioactive Compounds, Antioxidant Capacity, and Enzymes of Raspberries at Different Maturity Stages, Effects of Organic vs. Conventional Fertilization

**DOI:** 10.3390/foods10050953

**Published:** 2021-04-27

**Authors:** María Noemí Frías-Moreno, Rafael A. Parra-Quezada, Gustavo González-Aguilar, Jacqueline Ruíz-Canizales, Francisco Javier Molina-Corral, David R. Sepulveda, Nora Salas-Salazar, Guadalupe I. Olivas

**Affiliations:** 1 Faculty of Agrotechnological Sciences, Autonomous University of Chihuahua, Pascual Orozco Avenue, Campus 1, Santo Niño, Chihuahua C.P. 31350, Mexico; friasnohemi11@gmail.com (M.N.F.-M.); raparra@uach.mx (R.A.P.-Q.); nora.salas09@yahoo.com.mx (N.S.-S.); 2 Coordination of Food Technology of Plant Origin, Center for Research in Food and Development, A.C. (CIAD), Carretera Gustavo Enrique Astiazarán Rosas, No. 46, Hermosillo C.P. 83304, Mexico; gustavo@ciad.mx (G.G.-A.); jaruka89@gmail.com (J.R.-C.); dsepulveda@ciad.mx (D.R.S.); 3 Center for Research in Food and Development, A.C. (CIAD), Rio Conchos S/N Parque Industrial Apdo. Postal 781, Cuauhtémoc C.P. 31570, Mexico; javiermolina@ciad.mx

**Keywords:** anthocyanins, antioxidants, antioxidant enzymes, ascorbic acid, berries, farming systems, PAL, phenolic compounds, *Rubus idaeus* L.

## Abstract

Raspberries are important sources of bioactive compounds, whose synthesis is influenced by the fertilization system and the maturity stage. This study evaluated the effect of organic and conventional fertilization systems on raspberries at three maturity stages, pink, ripe, and overripe. Physicochemical characteristics, bioactive compounds (phenolic profile, vitamin C), antioxidant capacity (DPPH, FRAP, TEAC, and ORAC), phenolic-associated enzyme, phenylalanine ammonia lyase (PAL), and antioxidant enzymes (SOD, CAT, GPx, and APX) were evaluated. The physicochemical determination of the fruit did not reveal differences between fertilization systems. Regarding bioactive compounds, higher content of anthocyanins was found in organic raspberries at all maturity stages. Organic fertilization increased the content of ellagic acid and gallic acid at all stages of maturity. Higher content of caffeic, hydroxybenzoic, protocatechuic acid, and vitamin C was observed in organic raspberry at the overripe stage. Raspberries grown with organic fertilization exhibited higher values of antioxidant capacity by the DPPH, FRAP, and TEAC methods at all maturity stages. Raspberries under organic fertilization showed significantly greater activity of CAT, SOD, APX, GPX, and PAL. The present study suggests that organic fertilization induces oxidative stress causing an increase in antioxidant defense mechanisms, enhancing bioactive compound production, and improving antioxidant capacity in raspberries.

## 1. Introduction

Bioactive compounds from fruits and vegetables are highly valued by consumers due to their health properties that reduce the risk of contracting chronic diseases [1]. Raspberry fruits (*Rubus idaeus* L.) are considered an important source of bioactive compounds, particularly flavonoids such as anthocyanins and flavonols, phenolic acids (mostly ellagic and gallic acids), and vitamin C [2,3].

The synthesis of bioactive compounds can be influenced by different factors, such as the fertilization system and the maturity stage [4,5]. Fertilization plays an essential role in crop production, increasing yield, and improving quality [6]. However, there has been a growing concern about the negative impact that chemical fertilizers could have regarding environmental pollution, human health, and microbial damage.

On the other hand, the demand for organic food is increasing, as the consumer is looking for products free of agrochemicals. Organic products are also being promoted as more nutritious and healthier since it is considered that they could have a higher concentration of bioactive compounds; however, to date, the results have been contradictory. While, at the same time, some researchers find that organic fertilization increases bioactive compounds [7,8,9,10], other researchers find the opposite [11,12]. In the case of raspberry, while Jin et al. [13] showed that organic raspberry fruits present higher values in antioxidant capacity, specific flavonoid content, and activity of antioxidant enzymes, Sablani et al. [11] found no difference between organic and conventional management regarding the total content of anthocyanins, phenolic compounds, and antioxidant capacity.

Contradictory theories in both directions show logic and sustenance. On the one hand, one theory indicates that in organic systems the plant faces stress conditions that may induce the antioxidant defense mechanism that includes enzymes: superoxide dismutase (SOD), catalase (CAT), ascorbate peroxidase (APX), glutathione peroxidase (GPX), and non-enzymatic compounds such as ascorbic acid (AsA) and phenolics [14], the latter being synthesized by the activity of the enzyme phenylalanine ammonium lyase (PAL) [15]. Another theory focuses on exposing agrochemicals as the cause of abiotic stress in the plant, activating defense mechanisms and causing an increase in bioactive compounds and antioxidant activity [16].

Considering the number of contradictory results, more studies are necessary to better understand plants’ behavior to activate their defense mechanism [17]. Currently, the literature provides insufficient information on the systems used, particularly in the cultivation of raspberry [11]. Hence, it is essential to detail the agricultural practices used in both conventional and organic systems to better understand the effect of different cultivation techniques on bioactive compounds. Thus, the purpose of the present study was to evaluate the effect of the fertilization scheme, organic vs. conventional, on the antioxidant defense mechanism and the synthesis of bioactive compounds in Heritage raspberry; through the characterization and quantification of phenolic acids, flavonols, anthocyanins, vitamin C, oxidative enzymes (CAT, SOD, APX, and GPX), phenylalanine ammonium lyase (PAL) and antioxidant capacity. For a better understanding of the effect of these fertilization schemes, the study was carried out at three different maturity stages pink (under-ripe), ripe (red), and overripe (intense red).

## 2. Materials and Methods

### 2.1. Plant Material

The Heritage variety raspberry fruits were grown in the experimental field of the Faculty of Agrotechnological Sciences, Autonomous University of Chihuahua, Cuauhtémoc Campus (28°24′45.1″ N, 106°52′ 54.9″ W, and 2060 masl). The experiment was composed of 6 rows of 20 m each, with a distance between rows of 2 m. Each row was considered as one experimental unit. Two treatments, conventional and organic management, were considered (with 4 m between organic and conventional fields). The present study was carried out during the 2017 production, with precipitation of 539.5 mm, and average minimum and maximum temperatures of 6.8 °C and 24.0 °C, respectively.

### 2.2. Fertilization Scheme

Plants were fertilized by the direct addition of synthetic chemicals or organic products, supplying periods of vegetative growth, flowering, and production. The amounts of synthetic chemicals and organic products were calculated to end with similar total nitrogen content (~150 kg/ha). In the organic treatment, vermicompost, vermicompost leachate, and a commercial organic fertilizer were applied; their characteristics are indicated in Table 1.

Organic fertilization began in early May and continued weekly until August 15th, with 16 applications. The total amount of vermicompost applied was 15 t/ha, the total vermicompost leachate applied was 24 m^3^/ha (~24 t/ha), and the entire commercial organic fertilizer (GreenBackS11) was 7.5 t/ha. The final dose of minerals is shown in Table 1. For the conventional management treatment, synthetic commercial nitrogen, phosphorous, and potassium fertilizers were applied as ammonium nitrate, triple 17, and potassium sulfate with two applications per month, from May to August 15th (8 applications). The final dose of nitrogen, phosphorus, and potassium applied was 150 kg/ha, 40 kg/ha, and 190 kg/ha, respectively (Table 2). The fruits were harvested on August 17th, 2017 (two days after the last fertilization). Fruits were collected at three stages of maturity, which were classified as pink (under-ripe), ripe (red), and overripe (intense red).

After fertilization, soil nutrients were as presented in Table 3 (measurements done in August 2017).

### 2.3. Physicochemical Analysis

The raspberry fruits were analyzed for their physicochemical characteristics of weight, color, firmness, °Brix, titratable acidity and pH. The average weight of the fruits was obtained by dividing the total yield (g) by the number of fruits harvested per experimental unit. The color was determined using a Konica Minolta colorimeter (Spectrum photometer CM-600d), where L, a, and b values were observed (Cielab scale), and °hue was obtained [18]. To measure the firmness, a Brookfield C3T Texturometer was used, equipped with a 2 mm flat probe. Each fruit was compressed 2 mm at a speed of 0.5 mm/s, and the maximum force developed during the test was recorded in g force [19]. Total soluble solids, titratable acidity, and pH values were determined by AOAC [20]. For the physicochemical analyzes, six repetitions were performed. For the following methodologies, fruits were lyophilized, packed in vacuum-sealed Eppendorf tubes, and stored at −20 °C until their analysis.

### 2.4. Measurement of Phenolic Components (Phenolic Acids, Flavonols and Anthocyanins)

The method reported by Mattila and Kumpulainen [21] was used, with modifications described by Velderrain-Rodríguez et al. [22]. The monomers (aglycone form) of the polyphenols found within the fractions of raspberry extracts were quantified by ultra-high-performance liquid chromatography using a BEH C18, 130 Å, 1.7 µm, 3.0 mm × 100 mm column. A UPLC-DAD diode array detector at 270 nm (ACQUITY System, class H; Waters, Milford, MA, USA) was used. The mobile phases used were 0.5% formic acid and 80% methanol. The total run time was 30 min, the column temperature was set at 60 °C and a volume injected was 1 µL. All samples were filtered with 0.22 µm acrodisks before injection, considering six replicates in total. The results were interpreted using calibration curves with Sigma-Aldrich certified standards and expressed as mg/100 g of dry weight.

For the extraction of anthocyanins, the methodology reported by Abdel-Aal and Hucl [23] was followed. The identification and quantification of anthocyanins were carried out by the ultra-high-performance liquid chromatography technique with a UPLC-DAD diode array detector (ACQUITY System, class H; Waters, USA) at 520 nm, using a column ACQUITY UPLC BEH C18, 130 Å, 1.7 µm, 3.0 mm × 100 mm. The mobile phases were 2% formic acid and acetonitrile. The total run time was 30 min, the column temperature was set at 60 °C, and a volume of 5 µL was injected. All samples were filtered with 0.22 µm acrodisks before injection, considering six replicates. The results were interpreted using calibration curves with Sigma-Aldrich certified standards and were expressed as mg/100 g of dry weight.

### 2.5. Determination of Vitamin C Content

Ascorbic acid was assessed using a UPLC-DAD according to the method described by Odriozola-Serrano et al. [24] with some modifications by Robles-Sánchez et al. [25]. A lyophilized raspberry sample of 1 g was homogenized with 20 mL of a solution of metaphosphoric acid: acetic acid:distilled water (3:8:89). The mixture was filtered with a Whatman filter paper No. 1, centrifuged at 14,000 rpm for 15 min at 4 °C; finally, it was filtered with a 0.22 µm acrodisk. A 20 µL was injected into the de UPLC system. The results were expressed as mg of ascorbic acid/100 g of fresh weight (mg/100 g FW). Samples were analyzed using six replicates.

### 2.6. Measuring the Antioxidant Capacity DPPH, FRAP, TEAC and ORAC

Antioxidant capacity was evaluated by DPPH, FRAP, TEAC and ORAC assays. The DPPH assay was performed according to the method reported by Brand-Williams et al. [26] with some modifications as detailed by Palafox-Carlos et al. [27]. The stock solution was prepared by mixing 2.5 mg of DPPH radical with 100 mL of methanol. Subsequently, the solution was adjusted to an absorbance of 1.0 ± 0.02 at 515 nm. 20 µL samples of the extract (1:10 dilution) were placed on a microplate and 280 µL of DPPH radical was added. The mixture was kept in the dark for 30 min. The scavenging capacity was determined by the ability of the antioxidants to reduce the absorbance of the radical (515 nm) after the incubation time, using six replicates. The absorbance was read using a Microplate Reader (BMG Labtech Inc., Model FLUOstar Omega, Cary, NC, USA). Trolox was used as standard and methanol (80%) as a blank. Results were expressed as mg of Trolox equivalents (TE)/100 g of fresh weight. FRAP was determined in the sample extracts according to Benzie and Strain [28]. The method is based on the ability of the sample to reduce a ferric tripyridyltriazine (Fe^III^-TPTZ) to the ferrous form Fe^II^ that exhibits a blue color. So, absorbance was directly related to the reducing power of antioxidants. FRAP reagent (280 µL) was added to 20 µL of the extract (1:10 dilution) and kept for 30 min in the dark. The absorbance was read at 630 nm using Micro plate reader (BMG Labtech Inc., Model FLUOstar Omega, USA). Calibration curve was prepared using an aqueous solution of Trolox as standard. Results were expressed in Trolox equivalents (TE) per 100 g of fresh weight. TEAC value was determined according to Dávila-Aviña et al. [29], methodology derived from Miller et al. [30], and Re et al. [31]. The ORAC assay was performed according to Ou et al. [32], which is an improved method for oxygen radical absorbance capacity using fluorescein. The reaction mixture was prepared by mixing 25 µL of the sample with 150 µL of 10 nM fluorescein. The reaction was initiated by adding 25 µL of the AAPH radical (2,2′-azobis(2-amidinopropane) dihydrochloride, 240 mM). The decrease in fluorescence was measured every 90 s for 30 min at an excitation wavelength of 485 nm and emission wavelength of 520 nm in a micro plate reader (FLUOstar Omega). Phosphate buffer (75 mM, pH 7.0) was used as a blank, and serial dilutions of Trolox were used as a standard (6.25–200 mM). The results were calculated from the Trolox standard curve, and expressed as mg TE/100 g of fresh weight (FW).

### 2.7. Determination of Antioxidant Enzymatic Activity of CAT, APX, SOD, and GPx

Five grams of raspberry tissue were homogenized in 5 mL of 0.1 M Tris-HCl buffer (pH 7.8) containing 0.002 M EDTA-Na and 0.002 M dithiothreitol. The homogenate was centrifuged at 25,000× *g* for 20 min at 4 °C, and the supernatant was used for enzyme assays. Catalase activity (CAT, EC 1.11.1.6) was measured according to Beers and Sizer [33] with slight modifications. The reaction mixture consisted of 2 mL of sodium phosphate buffer (50 mM, pH 7.0), 0.5 mL of H_2_O_2_ (40 mM), and 0.5 mL of the enzyme. H_2_O_2_ decomposition was measured by decreasing absorbance at 240 nm. Ascorbate peroxidase activity (APX, EC 1.11.1.1) was tested according to Nakano and Asada [34]. The reaction was initiated by the addition of ascorbic acid, and the oxidation of ascorbate was measured through the absorbance at 290 nm. Enzyme activity was measured using the molar extinction coefficient for ascorbate (2.8 mM/cm). The activity of superoxide dismutase (SOD, EC 1.15.1.1) was based on the measurement of the autoxidation of epinephrine, which is inhibited by SOD. To 0.95 mL of 50 mM sodium carbonate buffer (pH 10.2), 50 µL of enzyme extract, and 50 µL of 10 mM epinephrine (Sigma-Aldrich, Toluca, Mexico) were added. The kinetic of the absorbance was measured for 10 min at a wavelength of 490 nm. A unit of SOD activity is the amount of enzyme required to inhibit the initial rate of epinephrine autoxidation by 50% in one minute [35]. The protein content in the enzyme extracts was determined with the Bradford method, using bovine serum albumin as standard. The specific activity of all enzymes was expressed as units per milligram of protein (U/mg protein). GPx was determined as described by Wendel [36].

### 2.8. Phenylalanine Ammonium Lyase (PAL, EC 4.3.1.24) Activity

Extraction of PAL was carried out following the method defined by Mori et al. [37], with some modifications described by Oliveira et al. [14]. The pulp samples (1 g) were homogenized for 3 min at 4 °C with a 2 mL buffer (0.1 M Tris-HCl pH 8.0), 1 mM EDTA, and 0.5 g of polyvinylpyrrolidone (PVP). The homogenate was centrifuged at 5000× *g* for 20 min. The supernatant was used to determine PAL. The reaction mixture contained 100 mM Tris-HCl buffer (pH 8.4), 40 mM L-phenylalanine and 100 µL of the enzyme to a total volume of 880 µL. The reaction was stopped by the addition of 6 M HCl and the absorbance was measured at 290 nm. PAL activity was expressed as µmol of cinnamic acid/mg protein/min.

### 2.9. Data Analyses

An experimental design with two factors was used, being factor A the fertilization management (organic and conventional) and factor B the stages of maturity (3 stages of maturity). Cultivar rows were considered as experimental units (six). The response variables were weight, color, firmness, °Brix, titratable acidity, pH, profiles of phenolic acids, flavonols and anthocyanins, vitamin C, antioxidant capacity and activity of enzymes CAT, APX, SOD GPX and PAL. The data were analyzed with the SAS package with ANOVA and the separation of means, using the Tukey test (*p* ≤ 0.05).

## 3. Results and Discussion

### 3.1. Physicochemical Parameters

The main characteristics of raspberry are defined by qualitative traits of size, firmness, color, flavor, and overall appearance [38]; these criteria are important for most consumers. The weight, soluble solids, titratable acidity, and pH in the present study did not show significant statistical differences between the organic and conventional fertilization systems in the three different maturity stages (*p* > 0.05) (Table 4). These results agree within previously reported ranges for red raspberries [18,39,40,41]. Firmness showed significant differences between the different maturity stages (*p* ≤ 0.05) (Table 4). This is because, during the fruit’s ripening, there is a decrease in the total content of pectin and the dismantling of structures of the primary cell wall and the middle lamella, which causes a softening of the fruit [42].

The color was also influenced by the maturity stage, showing a decrease in the Hue angle values, from 33.7 in the semi-mature stage to 27.7 in the over-mature stage, changing from intense bright red to dark red. The color of the raspberry is determined by pigments, mainly monomeric anthocyanins, which are found in the cells of the fruit [43], and the present work shows a considerable increase in the concentration of Cyanidin-3, 5-glucoside, cyanidin-3-glucoside, cyanidin chloride, and pelargonidin-3-glucoside as the development of fruit maturity progresses. The color and firmness of the fruit are fundamental to attract consumer interest. Hence it is important to understand the physiology and the dynamics of valuable phytochemicals throughout development and maturation to select the optimal maturity stage of the fruits produced with organic and conventional agriculture.

### 3.2. Raspberry Phenolic Components

Phenolic compounds are the main group of secondary metabolites produced by plants in response to stress; these compounds have an important antioxidant activity with potential benefit to human health [17]. Red raspberry is considered among the mayor fruit sources of phenolic compounds [1]. In general, the present work showed that raspberries from organic fertilization had higher total phenolic compound concentrations when compared to conventional raspberries by 116% in the pink stage, 61% in the mature stage, and 47% in the over-ripe stage (*p* ≤ 0.05), (Table 5). According to Straus et al. [44] and Frías-Moreno et al. [45], a decrease in nitrogen supply enhances the concentration of secondary metabolites such as phenolic compounds in beetroot and tomato. Although the same amount of nitrogen was applied in both fertilization systems (~150 kg/ha) (Table 1 and Table 2), organic fertilization presented a lower concentration of NO_3_ in soil when compared to conventional fertilization (19.7 vs. 34.27 kg/ha) (Table 3). According to Gutser et al. [46] organic fertilizers could release nitrogen fairly slowly. This limitation of nitrogen could increase plant stress leading to intensification of the production of antioxidant compounds.

Maturity stage also affected phenolic compounds; it was observed that as the stage of maturity advanced, concentration of total phenolic compounds increased (Table 5). The profile of polyphenols was characterized to a great extent by anthocyanin content, cyanidin-3, 5 diglucoside, pelargonidin-3-glucoside, cyanidin 3-glucoside, and cyanidin chloride. Following, by concentration were phenolic acids, mainly ellagic, gallic, and chlorogenic acids; and at lower concentration *p*-coumaric, caffeic, hydroxybenzoic, and protocatechuic acids. To a lesser extent, flavonols were also identified (kaempferol, rutin, and quercetin); all these compounds have been reported as characteristic phenolics of red raspberry [47,48,49].

#### 3.2.1. Phenolic Acids

Seven phenolic acids were identified in raspberries, ellagic, gallic, chlorogenic, *p*-coumaric, caffeic, hydroxybenzoic, and protocatechuic acids. The total sum of phenolic acids quantified varied with the stages of maturity and among the fertilization management systems (Table 5). As the raspberry maturity developed, the sum of phenolic acids increased in organic fertilization, while, in conventional fertilization, a decrease was observed (Table 5). Fertilization affected the biosynthesis of phenolic acids; organic raspberries showed ~9%, ~30%, and ~40% higher concentration of phenolic acids at the pink, ripe and overripe stage when compared to conventional raspberries (*p* ≤ 0.05). Sedrnicka-Tober et al. [50] found 31% higher concentration of phenolic acids in organic apples when compared to conventional apples in the three different apple varieties studied. The lowest phenolic acid concentration was observed in conventional raspberries at the overripe stage (~56 mg/100 g dry weight), while the highest phenolic acid concentration was observed at the same ripe stage in organic fruits (77 mg/100 g dry weight), (Table 5). Data reported by Zhang et al. [51] among different conventionally produced raspberry cultivars reported amounts of free phenolic acids that varied from 15.73 to 71.35 mg/100 g of dry weight, values similar to those in the present study.

Ellagic acid was the main phenolic acid identified in raspberries, followed by gallic acid; both found at significantly higher concentration in organic raspberries at the three stages of maturity, pink, ripe and overripe (*p* ≤ 0.05). Wang et al. [5] reported a decrease in ellagic acid in raspberries as the fruit ripen, and similar results were found here for raspberries under conventional fertilization. However, in organic fertilization, an increase in ellagic acid was observed as the degree of maturity progressed (Table 5). Xiao et al. [52] found that raspberry fruits showed values of 21 mg/100 g of dry weight, similar data as found in the present study for conventional management.

#### 3.2.2. Flavonols

The flavonols identified in Heritage raspberries, under both fertilization managements, were kaempferol-3-β-d-glucoside, rutin, and quercetin-3-glucuronide. Ripe organic raspberries presented higher amounts of flavonols when compared to conventional (Table 5), (*p* ≤ 0.05).

In general, the results showed a low concentration of flavonols in raspberry (Table 5). According to Hakkinen et al. [53], red raspberries have a very low proportion of flavonols. Jakobek et al. [47] found ~0.4 mg/100 g FW of flavonols in red raspberry (~4 mg/100 g DW), similar to ripe conventional raspberries in this work (4.31 mg/100 g DW). Kaempferol-3-β-d-glucoside was the predominant flavonol at pink and ripe stage, and it was found at higher amounts in organic raspberry when compared to conventional fruits (*p* ≤ 0.05) (Table 5). Ponder and Hallmann (2019) found that organic raspberries were characterized by a higher level of kaempferol [54]. Mitchel et al. [55] also observed a higher concentration of kaempferol in organic tomatoes in a 10-year comparative study. In contrast, Sablani et al. [11] found no effect of the agricultural production system on the concentration of kaempferol. Rutin was observed largely in the semi-mature stage, with no influence of organic or conventional fertilization on this flavonoid (*p* > 0.05). The same happened with quercetin-3-glucuronide and quercetin-3-β-d-glucoside, where organic and conventional raspberries behaved statistically the same (Table 5) (*p* > 0.05). The profiles of the individual flavonoids found are in agreement with those mentioned by Dragišić et al. [56] for conventional management. In general, with the exception of Kaempferol-3-β-d-glucoside, there were no significant differences in the amount and profile of flavonoids in organic and conventional fruits. These values are also in agreement with those reported by Granato et al. [57], and those of Dutra et al. [58] who worked with grape juice showing the similarity between organic and conventional.

#### 3.2.3. Anthocyanins

Anthocyanins are compounds that give the characteristic color to red raspberries, which are produced by the plant as an antioxidant response to biotic or abiotic stress [59]. Anthocyanins play an important role in human health, playing a vital role in cardiovascular illnesses, cancer and diabetes, among others [60]. The present study identified the following compounds: cyanidin-3, 5-diglucoside, cyanidin-3-glucoside, cyanidin chloride, pelargonidin-3-glucoside (Table 5). According to Beekwilder et al. [61] the main anthocyanins found in raspberry are cyanidin and pelargonidin glycosides. Anthocyanins were the bioactive compounds found in higher concentrations in the present work (Table 5). The sum of anthocyanins in organic and conventional raspberry fruits increased with advancing fruit ripeness, presenting values from 900 to 4784 mg/100 g DW in conventional raspberries, and from 2027 to 7041 mg/100 g DW in raspberries under organic fertilization (Table 5). Anthocyanin biosynthesis commences when ripening begins and continues throughout the ripening phase of growth [62]; and its concentration is dependent on the plants’ exposure to the sun [54].

In the present experiment, organic raspberries presented a higher concentration of anthocyanins at all maturity stages (*p* ≤ 0.05), with values higher by 225% in pink, 162% in ripe, and 147% in over-ripe, compared to raspberries under the conventional fertilization system (Table 5). In this regard, Ponder and Hallmann [54] also observed that raspberries under organic management presented a greater accumulation of total anthocyanins. Likewise, each of the identified anthocyanins: cyaniding-3, 5-diglucoside, cyanidin-3-glucoside, cyanidin chloride, pelargonidin-3-glucoside, exhibited a higher concentration in organic raspberry (*p* ≤ 0.05), (Table 5). Cyanidin-3, 5-glucoside was the main anthocyanin identified in this study. Previous studies have also reported cyanidin-3, 5-glucoside as the main anthocyanin present in raspberry fruits [13,52].

### 3.3. Vitamin C

**Figure 1:**
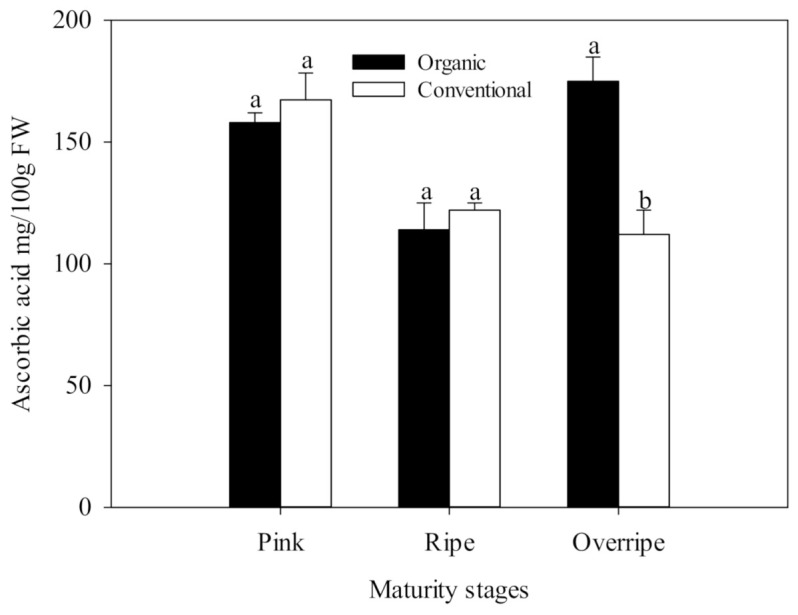
Ascorbic acid in three stages of maturity with two fertilization procedures. The results are represented as means ± standard deviation. Different letters between maturity stages indicate statistical difference at the 5% level of probability.

**Figure 2:**
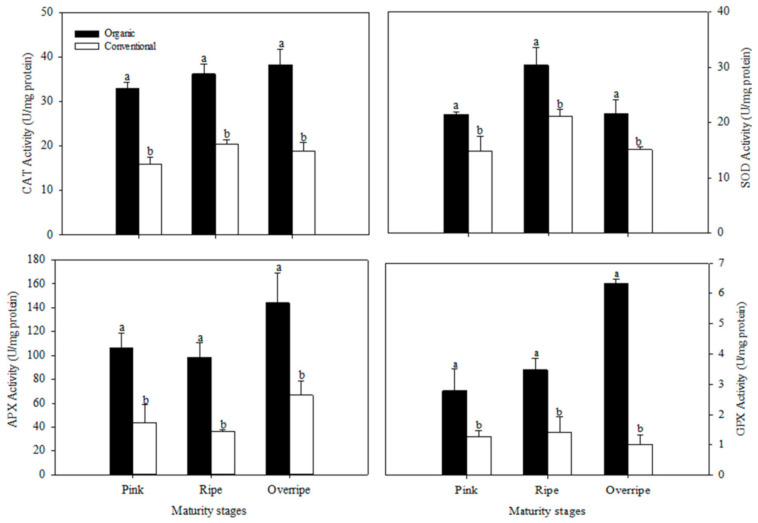
Effect of the fertilization system on catalase (CAT), superoxide dismutase (SOD), ascorbate peroxidase (APX), and glutathione peroxidase (GPX) in fruits at three stages of maturation. The results are represented as means ± standard deviation. Different letters between maturity stages indicate statistical difference (*p* ≤ 0.05).

The present study results show that the concentration of ascorbic acid presented statistical differences between the fertilization systems, only in the over-ripe stage of maturity (*p* ≤ 0.05) (Figure 1). This maturity stage in organic handling was characterized by having the highest concentration of Vitamin C with values of 174.90 mg/100 g fresh weight FW, which was significantly higher than conventional fertilization (125 mg/100 g FW), (Figure 1.). The differences found between organic and conventional raspberries may be associated with the oxidative stress due to insufficient availability of minerals in the organic cultivation system, generating a higher activity of the antioxidant metabolism of the plant and causing a higher production of ROS. Ascorbic acid is a cofactor for the antioxidant enzyme, ascorbate peroxidase (APX), which is particularly crucial in the defense against stress in plants [63]. In Figure 2, higher activity of the APX enzyme can be observed in the over-ripe stage with organic handling, which resembles the trend of the vitamin C results (Figure 1). The increase in APX activity suggests that organic fruits increased in H_2_O_2_, in advanced maturity, due to the participation of SOD. This enzyme eliminates the superoxide radical that catalyzes its conversion to H_2_O_2_, which is subsequently neutralized by catalase (CAT) and APX [10].

### 3.4. Antioxidant Activity

In this study, the antioxidant capacity of the raspberry samples at different stages of maturity was measured by several assays, ORAC, DPPH, TEAC, and FRAP, and results are presented in Table 6. Antioxidant capacity with the DPPH, TEAC, and FRAP showed higher values in the organic fertilization system, at all maturity stages (*p* ≤ 0.05). ORAC assay also presented higher antioxidant capacity on organic raspberry at ripe and overripe stage (*p* ≤ 0.05). However, ORAC assay showed no differences between organic and conventional fertilization at the pink maturity stage. The highest antioxidant activity was observed at ripe maturity stage in raspberries under organic fertilization, with values higher by 27% in DPPH, 46% in TEAC, 44% in ORAC 44%, and 66% in FRAP, when compared to conventional fertilization. Similar results have been found in other fruits such as strawberries, blueberries, and tomatoes [64,65].

Data are expressed as mean ± standard deviation. Results are expressed as mg TE/100 g fresh weight. Means in rows (at each maturity stage) followed by the same letter are not significantly different at the 5% level of probability. According to Straus et al. [44], a decrease in nitrogen supply, which is inherent in organic cropping, enhances the antioxidant activity in beetroot. This may be since, in organic agriculture, the nutrients are provided more slowly, becoming available to the plants in periods that vary from days to months [66]. Therefore, the low availability of mineral nutrients, mainly nitrogen, can cause stress to the plant, thus directing more resources to synthesize its chemical defense mechanisms. On the other hand, in conventional agriculture, the fertilizers that are applied are easily available for plants to direct their resources to plant growth, which results in a reduction in the production of secondary metabolites [17].

### 3.5. Antioxidant Enzymatic Activity

The antioxidant defense mechanisms in raspberry fruits include enzymes superoxide dismutase (SOD), catalase (CAT), glutathione peroxidase (GPx), and ascorbate peroxidase (APX). In the present work, the activity of CAT, SOD, APX, and GPx enzymes was higher in organic raspberries than in conventional ones, in all maturity stages (*p* ≤ 0.05) (Figure 2). Jin et al. (2012) evaluated the effect of fertilization systems on antioxidant metabolism and observed that the activities of CAT, SOD, and APX were higher in organic raspberries. Other investigations in different crops have also reported similar results, suggesting that the organic system stimulates the ability to eliminate free radicals [10,65,67]. The activity of the CAT, APX and GPx enzymes increased with increasing ripeness in raspberries of the organic system, presenting their highest activity in the overripe stage (Figure 2). The activity of the SOD enzyme also increased with maturity; however, it presented its highest activity in the mature stage (Figure 2). According to Tian et al. [68], reactive oxygen species (ROS) are implicated in the process of fruit senescence; as mitochondrial proteins are damaged by oxidative processes during senescence, a production of ROS is presented. This increase in ROS levels has been associated with the biosynthesis of antioxidant enzymes [69].

### 3.6. PAL Enzymatic Activity

**Figure 3:**
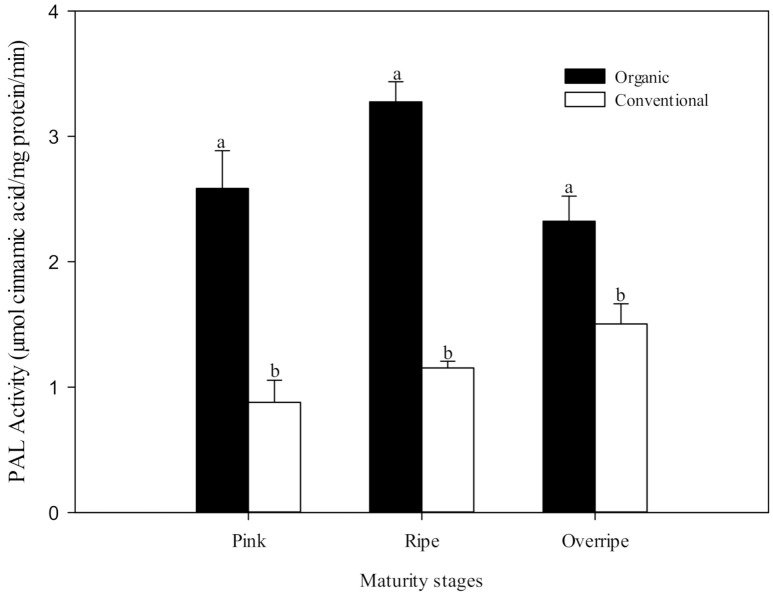
The activity of phenylalanine ammonium-lyase (PAL) at three stages of maturity with two fertilization procedures. The results are represented as means ± standard deviation. Different letters between maturity stages indicate statistical difference (*p* ≤ 0.05).

The first enzyme in the phenolic synthetic pathway is phenylalanine ammonia-lyase (PAL), which catalyzes the conversion of L-phenylalanine to trans-cinnamic acid, the initial step of the phenylpropanoid pathway (phenolic metabolism) in plants. PAL is a crucial enzyme in both plant development and defense since it controls a determining step of the speed of the biosynthetic pathway of phenolic compounds. According to Lima et al. [17] the activity of PAL is highly influenced by environmental factors. Several researchers have hypothesized that increased stress in plants, due to organic production techniques, can induce a higher activity of PAL and consequently higher levels of phenolic compounds [13,14,58]. The present study provides results that support this hypothesis. PAL activity was significantly higher (*p* ≤ 0.05) in organic fruits than in conventional ones, and this was found in all the maturity stages studied (Figure 3). This difference was also reflected in the content of phenolic compounds (Table 5), which, in general, showed a higher concentration in fruits grown under the organic system. Oliveira et al. [14] observed a higher PAL activity in organic tomatoes than conventional fruits (up to 140%) and a corresponding increase in total phenolics.

## 4. Conclusions

This work evaluated the influence of conventional and organic fertilization systems on oxidative/antioxidant metabolism and quality of red raspberry fruits at different maturity stages. The content of total bioactive compounds and the activity of the enzymes CAT, SOD, APX, GPX, and PAL were considerably superior in organic management, changing significantly during the maturation process. In general, in organic raspberry, as the maturity stage advanced, the sum phenolic compounds increased as well as the activity of the antioxidant enzymes CAT, APX, and GPX. Ripe raspberries with organic fertilization were characterized by the synthesis a higher content of ellagic acid, as well as presenting higher activity of SOD and PAL, and greater antioxidant capacity.

## Figures and Tables

**Table 1:** Chemical and physical characteristics of vermicompost, vermicompost leachate, and commercial organic fertilizer, and the amount of minerals applied (kg/ha).

	Vermicompost	Vermicompost Leachate	Commercial Organic Fertilizer *(GreenBack S11)*	Total Mineral Dose
pH	6.0	8.2	6.7	---
Organic material (%)	85	40	45	---
Total organic carbon (%)	18.57	-	-	---
N (%)	0.224	0.230	0.908	156.9 kg/ha
P (%)	0.012	0.030	0.772	66.88 kg/ha
K (%)	0.001	0.037	0.772	66.92 kg/ha
Ca (%)	0.133	0.001	5.22	411.69 kg/ha
Mg (%)	0.121	0.100	0.318	65.95 kg/ha

**Table 2:** Conventional fertilization applied (kg/ha).

Fertilizers	Active Ingredient	Dose Applied (Total)	Mineral Dose
Ammonium nitrate (NH_4_NO_3_)	35% N	315 kg/ha	110 kg/ha N
Triple 17 (N, P, K)	17% N, P, and K	240 kg/ha	40 kg/ha N 40 kg/ha P 40 kg/ha K
Potassium sulfate (K_2_ SO_4_)	53% K	284 kg/ha	150 kg/ha K

**Table 3:** Soil composition after fertilization treatments.

	Conventional Fertilization	Organic Fertilization
Soil type	loam	loam
Organic matter (%)	2.09	3.19
NO_3_ (kg/ha)	34.2	19.7
pH	6.8	7.6
P (ppm)	90.3	51.3
K (ppm)	474.5	388
Ca (ppm)	2712	3408
Mg (ppm)	354	444
Na (ppm)	187	182
Fe (ppm)	29.6	21.0
Zn (ppm)	5.8	6.4

**Table 4:** Physicochemical composition of raspberry in three stages of maturity with two fertilization systems.

Physicochemical Composition	Stages of Maturity					
	Pink		Ripe		Over-Ripe	
Fertilization System	Organic	Conventional	Organic	Conventional	Organic	Conventional
Weight (g)	3.01a	3.04a	3.05a	3.06a	3.0a	3.01a
Color (°hue)	33.75a	33.69a	30.45b	30.74b	27.54c	27.94c
Firmness (g force)	127.6a	124.8a	93.1b	91.6b	66.2c	65.9c
Soluble solids (°Brix)	10.9a	10.5a	11.0a	10.8a	11.3a	11a
Titratable acidity (g of citric acid/100 g of FW)	1.91a	1.92a	2.0a	1.92a	2.0a	2.0a
pH	3.05a	3.03a	3.08a	3.04a	3.13a	3.08a

Data are presented as means, *n* = 4. Means in rows (at each maturity stage) followed by the same letter are not significantly different at the 5% level of probability.

**Table 5:** Polyphenols profile (mg/100 g dry weight DW) in raspberry fruits at three maturity stages, pink, ripe, and overripe, grown under organic or conventional fertilization.

Phenolic Compounds Profile	Maturity Stages					
	Pink		Ripe		Over-Ripe	
	Organic	Conventional	Organic	Conventional	Organic	Conventional
Ellagic acid	28.47a	25.44b	38.80a	22.01b	27.74a	23.27b
Gallic acid	25.46a	22.68b	19.94a	17.52b	24.40a	16.61b
Chlorogenic acid	4.62b	7.23a	9.84a	10.21a	11.40a	7.34b
*p*-coumaric acid	4.24a	4.38a	2.77a	2.95a	3.64a	2.72b
Caffeic acid	2.87a	2.47a	4.97a	5.14a	6.23a	4.06b
Hydroxybenzoic acid	3.74a	1.27b	0b	1.01a	2.66a	0.79b
Protocatechuic acid	0.84a	1.00a	1.84a	1.49a	2.10a	0.93b
**Total phenolic acids**	**70.24a**	**64.47a**	**78.16a**	**60.33b**	**78.17a**	**55.72b**
Kaempferol-3-β-D-glucoside	4.71a	2.48b	2.035a	0b	0a	0a
Rutin	2.50a	2.81a	1.45a	1.66a	1.67a	1.42a
Quercetin-3-glucuronide	1.61a	1.80a	1.79a	1.74a	0.98a	0.86a
Quercetin-3-β-D-glucoside	1.95a	1.98a	0.94a	0. 91a	1.44a	1.23a
**Total flavonols**	**10.77a**	**9.07a**	**6.22a**	**4.31b**	**4.09a**	**3.51a**
Cyanidin-3, 5-diglucoside	1063.65a	373.82b	2540.03a	1522.72b	3150.13a	2187.31b
Cyanidin-3-glucoside	295.13a	88.19b	1103.62a	666.22b	1596.79a	907.25b
Cyanidin chloride	162.90a	103.36b	428.30a	277.23b	493.57a	375.12b
Pelargonidin-3-glucoside	505.33a	335.32b	1548.35a	1001.09b	1800.61a	1315.25b
**Total anthocyanins**	**2027.01a**	**900.69b**	**5620.30a**	**3467.26b**	**7040.97a**	**4784.93b**
**Total phenolic compounds**	**2108.02a**	**974.23b**	**5704.68a**	**3531.90b**	**7123.23a**	**4844.16b**

Data are presented as mean, *n* = 4. Means in rows (at each maturity stage) followed by the same letter are not significantly different at the 5% level of probability.

**Table 6:** Antioxidant capacity of raspberry fruits in three stages of maturity with organic versus conventional fertilization system.

Antioxidant Capacity	Maturity Stages					
	Pink		Ripe		Over-Ripe	
Fertilization systems	Organic	Conventional	Organic	Conventional	Organic	Conventional
DPPH	403.94 ± 3.33a	337.21 ± 19.39b	563.60 ± 22.81a	441.16 ± 12.38b	412.50 ± 22.53a	363.20 ± 16.46b
TEAC	295.41 ± 23.13a	242.14 ± 25.66b	422.59 ± 48.66a	289.82 ± 17.32b	356.51 ± 13.87a	219.27 ± 43.22b
FRAP	440.60 ± 28.4a	281.76 ± 17.78b	606.71 ± 23.44a	363.80 ± 10.62b	432.91 ± 41.99a	264.56 ± 28.44b
ORAC	20.84 ± 1.89a	20.68 ± 1.39b	38.75 ± 4.40a	26.79 ± 2.19b	35.10 ± 1.93a	27.42 ± 1.93b

## Data Availability

Data is contained within the article.
